# *The Organon* and Materia Medica Club of the Bay Cities of California

**Published:** 1896-08

**Authors:** W. E. Ledyard

**Affiliations:** Secretary


W. E. LEDYARD, *Secretary.*

Reported by Eleanor F. Martin, M. D.

---

### *THE ORGANON* AND MATERIA MEDICA CLUB OF THE BAY CITIES OF CALIFORNIA.

The regular semi-monthly meeting was held at the office of Dr. George H. Martin, 606 Sutter Street, San Francisco, Friday evening, April 3d, 1896.

Members present: Drs. J. M. Selfridge, A. McNeil, M. T. Wilson, M. F. Underwood, G. J. Augur, W. E. Ledyard, and George H. Martin.

The meeting was called to order at 8.10 o'clock by the President, Dr. J. M. Selfridge. The minutes of the previous meeting were read by the Secretary, and, after a correction by Dr. Underwood, were approved.

By request of Dr. McNeil, Section 80 of *The Organon* was read instead of 96.

### DISCUSSION.

Dr. McNeil—Hahnemann does not mention *all* chronic diseases in this section. He brings in a long list of chronic diseases which are psoric, but there are many chronic diseases that are not psoric. Chronic diseases that are cured by non-antipsoric remedies are not psoric diseases.

Dr. Selfridge—You mean to say, then, that Hahnemann's statement in regard to diseases which take their origin in psora is not correct?

Dr. McNeil—He does not give *all* chronic diseases here. We all cure some chronic diseases with non-antipsoric drugs, and these diseases do not originate in psora.

Dr. Selfridge—We had this same subject to discuss once before, and the point I made then I make now, and that is, that any remedy that cures a chronic disease is an antipsoric remedy even if Hahnemann has not mentioned it as such.

Dr. McNeil—Non-antipsoric remedies will cure chronic diseases arising from various causes, but will not cure those which come from a psoric condition. If you are right then it would not be necessary to make any distinction between antipsoric and non-antipsoric remedies.

Dr. Augur—Hahnemann mentions in the note to this section other chronic diseases.

Dr. Underwood—What does Hahnemann mean when he says that when prescribing for a chronic disease we must give an antipsoric remedy?

Dr. McNeil—He means then psoric diseases. The position that Dr. Selfridge takes is that Hahnemann did not know an antipsoric remedy from any other.

Dr. Martin—If we continue reading we will find that Hahnemann gives other chronic diseases besides those he mentioned in this last section. He lays the foundation and fundamental cause of all chronic diseases to three different sources, psora, sycosis, and syphilis.

Dr. McNeil—I want to know if there is any one here who has not cured chronic diseases with a single antipsoric remedy, as for instance rheumatism with *Rhus*, dysmenorrhœa with *Pulsatilla*, etc.?

Dr. Martin—I do not think that I ever cured a chronic disease with one remedy alone.

Dr. McNeil—I have cured many of them. One case in particular of a young girl suffering from dysmenorrhœa whom I cured with *Pulsatilla.*

Dr. Augur—I have always understood that *Pulsatilla* was an antipsoric remedy.

Dr. McNeil—*Ignatia*, *Aconite*, *Pulsatilla*, and *Rhus-tox.* are classed as non-antipsorics in the *Materia Medica Pura.*

Dr. Selfridge—If I understand Hahnemann, he classes all chronic diseases as coming from one of three miasms, psora, sycosis, or syphilis, and if he does not mean this I should like to know where he says that they do not.

Dr. McNeil—In every case of rheumatism for which *Rhus* is the remedy, it is usually a rheumatism arising from dampness, often in out-of-door laborers with unwholesome habits of living. Chlorosis, which is amenable to *Pulsatilla*, is usually caused from improper living. Nervous diseases cured by *Aconite* often arise from fear. *Ignatia* is the remedy for long protracted grief, etc. These conditions are certainly not psoric conditions.

Dr. Selfridge—And you call these chronic diseases? Is fright a chronic disease?

Dr. McNeil—Chronic diseases often arise from fear.

Dr. Selfridge—Rheumatism is not chronic unless it is based upon psora, sycosis, or syphilis. A case of rheumatism from exposure will run its course, unless by frequent exposures it be kept up for some time. Any remedy that will cure a pure, clear-cut, chronic case is an antipsoric remedy.

Dr. McNeil—Chronic diseases arising from fright or grief are not psoric diseases, and they are cured by non-antipsoric remedies.

Dr. Selfridge—The patients in which your chronic diseases arise from these causes are subjects of one of the three miasms I mentioned.

Dr. Martin—That is just exactly it. There is back of all these symptoms the psoric condition. Hysteria is never pure and simple without some exciting cause; also madness; but back of the exciting cause is the predisposition to it which is the psora, and which has now been aroused.

Dr. McNeil—A young lady suffering from dysmenorrhœa, narrated her case with tears. She had suffered intensely with occipital pains. Her father was a locomotive engineer, and he would have to rub her occiput with all the strength of his hands in order to relieve her at all. *Pulsatilla* made a perfect cure in this case. It is a non-antipsoric remedy.

Dr. Underwood—*Pulsatilla* is an anti-sycotic remedy. It cures figwarts.

Dr. McNeil—*Aconite* and *Ignatia* are not antipsoric remedies and they cure chronic diseases.

Dr. Underwood—I would like to know where we can go to-day and find a person who is not affected with psora.

Dr. Selfridge—There is no one who is not affected.

Dr. McNeil—For the effects of grief, *Phosphoric-acid* or *Natrum-muriaticum* may be the remedies.

Dr. Selfridge—How do you know that in the case you cured with *Pulsatilla* there was not present either psora, sycosis, or syphilis?

Dr. McNeil—It is not necessary for me to know it.

Dr. Selfridge—How did Hahnemann know or how do we know how many antipsoric remedies there are?

Dr. Selfridge then read from *The Organon*, Sections 96 and 97.

DISCUSSION.

Dr. McNeil—I like to treat children because they do not lie. Patients who have reached years of discretion always lie. Some exaggerate because they want to think they are very sick. There is another class of patients in whom every point cannot be brought out. Florence Nightingale said that there were two classes of patients, those who exaggerate and the heroic. The latter do not like to complain at all. I had such a case, a young man with typhoid fever. I could hardly get a symptom out of him but those I could see.

Dr. Underwood—I notice that patients who come into my office who do not give me the symptoms, I give a dose of *Sac-lac.* and divert their attention, and after a short time they tell me all about themselves.

Section 98 was read.

DISCUSSION.

Dr. Ledyard—In regard to what Dr. Underwood said: I have often given the remedy I supposed to be the correct one, and they then commenced to talk and I have wished that I had not given them that remedy.

Dr. McNeil—I think I learn more in examining patients as I grow older. We cannot reach Hahnemann's standard in this regard except by hard study and observation.

Sections 99 and 100 were read.

### Discussion.

Dr. McNeil—Do these diseases just mentioned form exceptions to what he has stated before?

Dr. Selfridge—I don't think they do. I have treated a great many cases of small-pox, and have rarely found two epidemics alike.

Dr. Ledyard—This is so in whooping-cough.

Dr. McNeil—In a case of croup I found the mother fanning the child. I cured with *Carbo-vegetabilis*, from *desire to be fanned. China wants to be fanned slowly.*

Dr. Underwood—*Medorrhinum* wants to be fanned fast, *especially upon the hands.*

Sections 101–103 were read.

### Discussion.

Dr. Underwood—Hahnemann says here *chronic miasmatic diseases*, showing that he meant there were other chronic diseases as well.

Dr. McNeil—He means here the distinction between miasmatic and epidemic chronic diseases.

The President then suggested that the vacancy in the Vice-Presidency should be filled, whereupon Dr. Martin moved that Dr. McNeil be appointed to that office. Seconded by Dr. Wilson and carried.

The meeting then adjourned, to meet again the third Friday in April at the office of Dr. J. M. Selfridge, in Oakland, when the reading of *The Organon* would be commenced at Section 104.

